# A Survey of Blockchain Enabled Cyber-Physical Systems

**DOI:** 10.3390/s20010282

**Published:** 2020-01-03

**Authors:** Heena Rathore, Amr Mohamed, Mohsen Guizani

**Affiliations:** 1 Department of Computer Science, University of Texas, San Antonio, TX 78249, USA; 2 Department of Computer Science, Qatar University, Doha 2713, Qatar; amrm@ieee.org (A.M.); mguizani@ieee.org (M.G.)

**Keywords:** healthcare, smart grids, survey, transportation, industrial control systems, Iot, internet of things, cyber-physical systems, bitcoin, blockchain

## Abstract

Cyber-physical systems (CPS) is a setup that controls and monitors the physical world around us. The advancement of these systems needs to incorporate an unequivocal spotlight on making these systems efficient. Blockchains and their inherent combination of consensus algorithms, distributed data storage, and secure protocols can be utilized to build robustness and reliability in these systems. Blockchain is the underlying technology behind bitcoins and it provides a decentralized framework to validate transactions and ensure that they cannot be modified. By distributing the role of information validation across the network peers, blockchain eliminates the risks associated with a centralized architecture. It is the most secure validation mechanism that is efficient and enables the provision of financial services, thereby giving users more freedom and power. This upcoming technology provides internet users with the capability to create value and authenticate digital information. It has the capability to revolutionize a diverse set of business applications, ranging from sharing economy to data management and prediction markets. In this paper, we present a holistic survey of various applications of CPS where blockchain has been utilized. Smart grids, health-care systems, and industrial production processes are some of the many applications that can benefit from the blockchain technology and will be discussed in the paper.

## 1. Introduction

Cyber-physical systems (CPS) are an architectural paradigm coupled with pervasive sensing and communication technologies to provide multiple benefits to the economy and society. In other words, it is an engineered system where the physical system or process is augmented with cyber components, such as computational hardware and communication network [1]. These components are very tightly integrated with each other, which means the functionality of one component is dependent on the other component. CPS has seen exponential growth in recent years in areas, such as energy, health, transportation, and the Industrial Internet of Things (IIoT). Key areas of research, while designing such systems to be smart, efficient, and flexible, are stability, reliability, robustness, security, and privacy. However, rapid advancements in the enabling technologies, have also exposed such systems to serious and profound risks. If such risks are not managed, we would lose the incredible benefits that they can provide. Blockchain has a great potential to create new foundations for most distributed systems by efficiently establishing trust among nodes. It is a fundamental technology to enable decentralization and play an important role in CPS domain.

Blockchain is a secure digital ledger of transactions that can be configured to record, not only transactions in the financial world but also in other areas where maintaining historical evidence of the transactions has value. It is the key technology behind bitcoins, a type of cryptocurrency. Bitcoins were developed after the financial crisis of 2009 as an alternative to traditional currency [2]. It is widely considered that one of the many reasons behind this crisis was the single point of failure, exemplified by how centralized banks maintained financial records. There was no oversight in this process, hence the lack of fault-tolerant checks and balances. Financial institutions, for a long time, have talked about the need for distributed decision-making process, but not acted on it, till the advent of crypto-currencies fueled by the blockchain technology. A group of anonymous hackers, with the alias of Satoshi Nakamoto, were responsible for writing the first set of code. It is a type of mechanism which validates, verifies, and confirms the transactions by recording them in a distributed ledger of blocks. It implements a consensus protocol to arrive at an agreement on the validity of a transaction by creating a chain of blocks. This immutable chain of blocks is trusted and verified, thereby making them a highly secure mechanism for maintaining a distributed ledger of transactions.

Initially, the blockchain technology was primarily utilized for protecting the financial transactions, smart contracts, storage systems, and notary. However, its benefits were soon recognized by other applications, such as supply chain, healthcare, transportation, and energy, as the industry realized that it can improve efficiency by adopting blockchain. This has spawned an active area of work, wherein researchers and scientists are now looking at other applications where this technology can be utilized. Energy, health and transportation are some of the most commonly cited applications. This key contributions of this paper are:

- Provide a holistic survey of applications where blockchain is being utilized in CPS systems.
- Describe applications involving a confluence of communications, sensing, and computation as opposed to cyber-only systems.
- Provide a mathematical formulation to analyze when distributed ledger techniques, such as blockchain, are useful for a particular application.

The overall organization of the paper is: Section 2 explains the core concepts of blockchain technology. Section 3 presents the blockchain applications in CPS systems, such as healthcare, industrial control systems, and transportation and smart grids. Section 4 presents blockchain limitations and provides future directions in another next generation cryptocurrency, namely IOTA. Section 5 entails and provides a mathematical model to determine if various application domains could benefit from blockchain. Section 6 concludes the paper.

## 2. Blockchain Technology

The hacking of over a billion Yahoo accounts [3], the Equifax data breach [4], and increased ransomware attacks [5] are just a few of many reported cyber attack incidents in recent years. As a matter of fact, over one million cyber threats are released every day and by 2020, over 200 million IoT devices [6] will need security. Some industry experts anticipate this number to reach 29 billion in the next couple of years. Blockchain, a distributed system to manage transactions, which uses consensus among network participants to build trust, is being considered as a viable alternative to protect against cyber-attacks. Such distributed systems have many advantages as compared to centralized systems, which fail to scale as the number of connected devices increases. In [7], authors outlined how a chain of cryptographically secured blocks can be used to preserve and protect the integrity of past information [7]. The idea of proof-of-work was established in 1993 as a countermeasure to the proliferation of spam and other system abuses. Later in 2008, a white-paper [8] was released which established the foundation of bitcoin, a cryptocurrency based on the concept of blockchain. This triggered new research in this emerging topic that achieved significant milestones, such as the adoption of smart contracts based ethereum [9], application in the financial industry, and mention in the Harvard Business Review as a basic and fundamental innovation for financial industries. Today, there is a robust and fast-growing ecosystem encompassing blockchain, and an increased number of applications are migrating towards a decentralized approach for securing transactions. While blockchain was originally developed for cyber-only applications, over time, applications that combine both cyber and physical aspects are also benefiting from this concept.

### 2.1. Blockchain Explanation

A blockchain is an immutable distributed database to which new time-stamped transactions can be appended and grouped into a hash-chain of blocks [10]. The underlying blockchain protocol defines how multiple copies of such blocks can be constructed and maintained in a distributed fashion. A key aspect of this protocol is deciding how a network of participants, known as miners, can establish consensus on the current state of the blockchain. This algorithm assumes that, in any given time epoch, only a fraction of the miners could turn malicious or faulty. There are different types of blockchain architectures (i.e., public, private, permissioned, and permission-less). A public blockchain is one that allows anyone to join. They are usually permission-less where all the users have equal rights. A private blockchain is a closed blockchain where privacy is important. Here, every participating node is pre-selected and vetted. They are permissioned and the users do not have equal rights in the network. One of the first, and still popular, permission-less blockchain protocol is bitcoin [8]. Every 10 minutes, on average, it selects a new miner in an unbiased fashion who then gets the right to commit or append a new block to the blockchain. The key question to be determined is who adds the next chain of transactions and how is it added. There are two prevailing strategies for the same, namely Proof of Work (PoW) and Proof of Stake (PoS). In simple terms, consider a situation where P1 wants to pay P2. P1 first announces its intent and then provides authenticity by signing the transaction using a cryptographic key. The Network operators, or miners, validate the authenticity of the digital signatures and availability of assets. Once these tasks are complete, the new transactions are added to the blockchain. Each block contains a unique code called a hash, which also contains the hash of the previous blocks in the chain, and is used to connect the blocks together in a specific order. Any miner has to perform a set of computations to establish their credibility as a leader. These computations solve a puzzle to map arbitrary size data to a fixed size. In any network, a leader can be chosen in one of these two ways. In Proof of Work (PoW), many miners try to solve the puzzle and the one that finishes first, broadcasts to the group proof that the work is done. Other miners then validate that the work done is correct. Once everyone confirms this, they select that particular miner as the leader. This approach is computationally expensive because many miners are trying to solve the puzzle simultaneously, until one of them succeeds.

**Figure 1:**
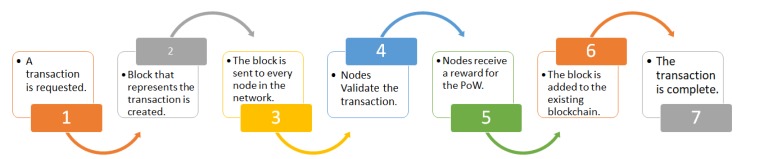
Blockchain process.

The typical top level view of blockchain is shown in Figure 1. Here, once the transaction is requested, a data structure for keeping the set of transactions is distributed to all nodes in the network. All the nodes perform the block verification process before adding anything to the blockchain structure. Once the nodes do the block verification, they receive reward for the proof of work. Likewise, each new node joining the distributed system of blockchain gets a full copy of the blockchain. When another block is made, it is sent to every node inside the blockchain framework. At that point, every node confirms the block and checks whether the data expressed there is right. If everything is correct, the block is added to the local blockchain in every node.

**Figure 2:**
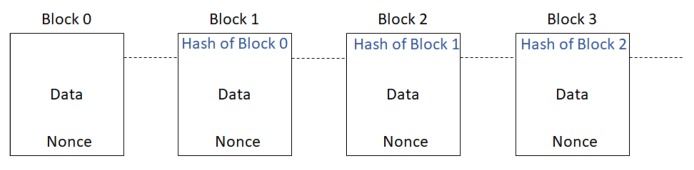
Blockchain: A chain of blocks where each node references the previous block. POW = Proof of Work.

The second method of doing this is called PoS. In this method, a leader, who has the highest amount of stake in the network, is selected. The amount of stake in the network is determined by the number of coins that the miner owns. This is based on the theory that the miner with a lot of stake in the network is most likely, to be honest. The rest of the network then implicitly accepts this leader by attaching its block to the leaders’ block. This maintains the longevity of consensus in bitcoin. The protocol also defines a reward mechanism as PoW involves significant computation, which also leads to one of the significant shortcomings related to scalability and transaction throughput. Figure 2 shows the illustration of transaction records of blockchain.

The primary purpose of the block is to maintain a list of verified transactions using a cryptographic hash function. The hash function is efficient because of the following properties:

- It generates an output of fixed length irrespective of the length of the input.
- It is deterministic which means that it generates the same output for a given input.
- It is irreversible which means that getting the same input from the output is not possible.
- Any slight perturbations to the original input generate new output.
- The hash computations are fast with minimal overhead.

**Figure 3:**
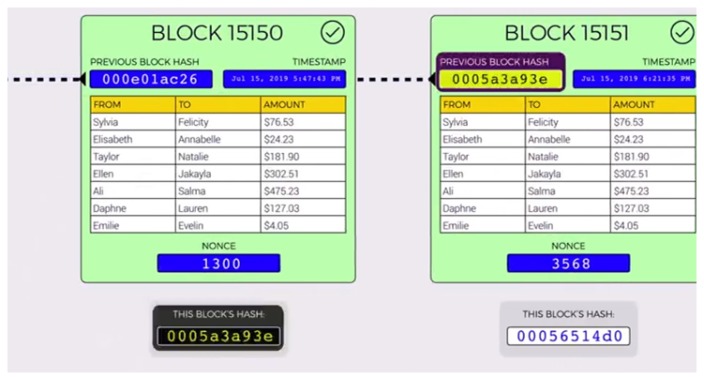
Chain of blocks.

The blocks in the blockchain are linked to the very first genesis block and are verified by the hashes. All the blocks are connected through the relationships of all their hashes, which means each block contains the previous hash, and these get further hashed in the next block. Any changes to the hash cause the chain to be broken because the original hash is still attached to the next block in the chain. Recalculating the original hash to restore the chain requires an enormous amount of computing power. In addition, nonce is added so that the miners can play with the data to produce a hash which outputs three leading zeroes, as shown in Figure 3. Once the miners have found a nonce that results in their block’s hash being below the difficulty threshold, the block is finally considered valid, and it can be broadcast to the network with that miner taking a reward for their effort.

A possible attack scenario in such a chain is that an attacker can alter the contents of the database and creates another chain of records by producing another set of transaction records. However, the action of changing anything in the chain has a domino effect, thereby invalidating all the blocks that follow. If a transaction on the chain is altered by a hacker, it invalidates the entire block, thereby requiring the network miners to repeat the task of finding a nonce that yields a hash value below the target difficulty. This makes the blockchain as the most revolutionary technology that is not only efficient but also the most secure among all the other state of art technologies.

Public blockchain architectures, such as bitcoin and ethereum, are open source and are permission-less. These types of blockchain architectures allow anyone to download the code, demonstrate proof of work, and earn the right to validate the transactions in the network. This type of architecture is open and transparent. Private blockchain architecture on the other hand, examples of which are R3 [11] and EWF [12], operate under the leadership. It is a type of semi-distributed architecture with permissioned read and/or write authority. This type of architecture is faster and has pre-approved participants with known identities.

### 2.2. Understanding Blockchain Using Financial Transaction as An Example

**Figure 4:**
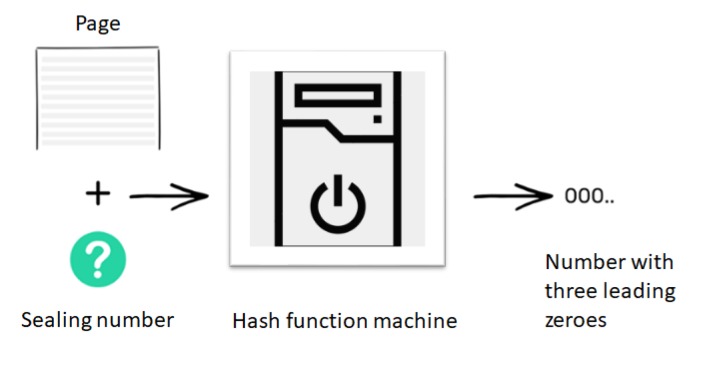
Hash function machine generating sealing number.

Normally, whenever two people want to transfer money among each other, they require a centralized authority, such as a bank, to manage the transactions entered in the bank logs, managed as a database. In other words, to establish trust between two people who typically know each other, we depend on an external third party, such as a bank. In order to avoid this, the concept of blockchain came into the picture. Consider, a situation, wherein there are ten individuals who do not want to use a bank to record the exchange of currency amongst themselves. They mutually agree to have constant access to each other’s accounts, without knowing the other’s identity. To start with, everyone has an empty folder. As time progresses, each of these 10 individuals will add transactions to their folder and a historical record of these transactions is maintained on a ledger. Let us suppose that person number 2 wants to send $10 to person number 9. To make the transaction, everyone checks whether person number 2 has sufficient balance to transfer $10 to person number 9. If she does, everyone makes a note of the transaction on their blank page. Transactions keep happening within the network and everyone keeps writing them down until their pages get filled. When this happens, everyone puts the page away in their folders, bring out a new page and start the whole process over again. The magic of blockchain lies when the page has to be put away in the folder. The deal is that when the page goes in the folder, everyone’s version of the page must look the same and it must not be modified ever in the future. To accomplish this, everyone needs to seal the contents of the page, which is accomplished by using the hash function, as described earlier. In this case, the hash function outputs a number with three leading zeros and it does so by trying various inputs. Thus, to seal a page containing a list of transactions, we need to figure out a number, which when appended to the list of transactions and fed to the machine, gives a code that starts with three leading zeros, as shown in Figure 4.

**Figure 5:**
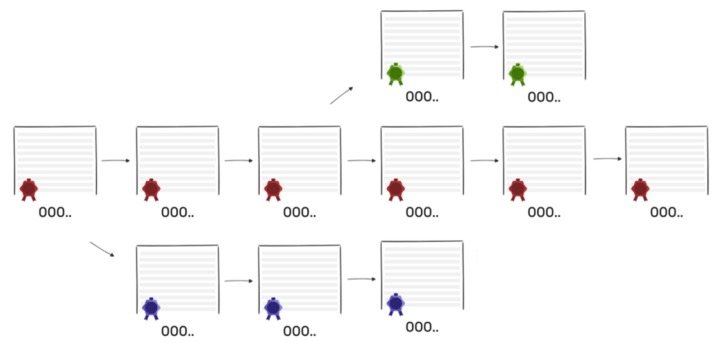
Longest chain in the network is the honest chain. Red is the honest chain. Green and blue are dishonest chains.

This step is done when there is no more room on the page to add new transactions. A sealing number for the page is calculated by everyone in the network. The network participant who figures this out first announces the sealing number to other members of the network. Once this event happens, all the other network participants verify that the hash number announced is valid. If it is, then the participant is chosen as a miner and everyone else seals their page with this same number and places it in their folder. In addition, every page in the blockchain depends on its previous page. If a hacker tries to modify a historical page, then the contents and sealing number of all subsequent pages would have to be modified in order to keep the chain consistent. This process of adjusting several pages and calculating new sealing numbers is time-consuming and provides a strong deterrent to doing so. Figure 5 shows if the hacker tries to modify the content the chain is shorter than the original chain. This is on the because from the page the untrustworthy person attempts to cheat, he would make another chain in the system, however that chain be unable to make up for becoming the legit chain—simply in light of the fact that one person’s speed can’t beat combined speed of other people in the network, hence guaranteeing that the longest chain in a network is the honest chain. In addition, other members of the network are quickly alerted about a potential threat from one of the members in case this happens.

### 2.3. Benefits of Blockchain

Blockchain has several advantages, such as it is one of the most secure ways of recording and authorizing information stored on the network. It is also a transparent storage mechanism where anyone on the network can verify the authenticity of the information. Furthermore, the data that is stored on the network cannot be changed without incurring huge overheads, which makes it secure and efficient. Blockchain transactions typically contain a peer-based proof, either of validity or authorization, instead of relying on a centralized application as an enforcer of constraints. It is a type of replicated and shared that is synchronized across members of the network. It records the transactions, such as the exchange of assets or data, among the participants in the network. It acts as a consensus mechanism ensuring that nodes that independently verify and process transactions stay in sync. There are some differences between blockchain and traditional centralized databases which are listed below:

- Since transactions propagate between nodes in a peer-to-peer fashion, blockchain uses a public-private cryptography scheme, such as Elliptic Curve Digital Signature Algorithm (ECDSA) [13], to digitally sign each transaction. However, it is computationally expensive to generate and verify these signatures. Additionally, due to the lack of sufficient randomness during the signature process, a hacker can recover the user’s private keys, thereby making the scheme more prone to attacks, especially since it is done by all the peers [14].
- In a distributed database, arriving at consensus among network members is a computationally intensive effort. Additionally, it also involves significant back-and-forth communication, depending on the consensus mechanism used. The consensus mechanism has 51% vulnerability, which means a single miner with more than 50% of the total hashing power can unilaterally launch an attack. This is practically impossible when the network size is large. Such attacks are less likely in a centralized database, even though they also have to contend with conflicting and aborted transactions.
- Whereas there is some level of redundancy in a centralized database, it is far less as compared to a blockchain which must process every transaction by every node independently to achieve better security and transparency.

## 3. Blockchain Applications for Cyber-Physical Systems (CPS)

With the growth in acceptance of computers over the past few decades, records have mostly migrated from being physical paper documents to digitized versions, created and managed on a computer.

**Figure 6:**
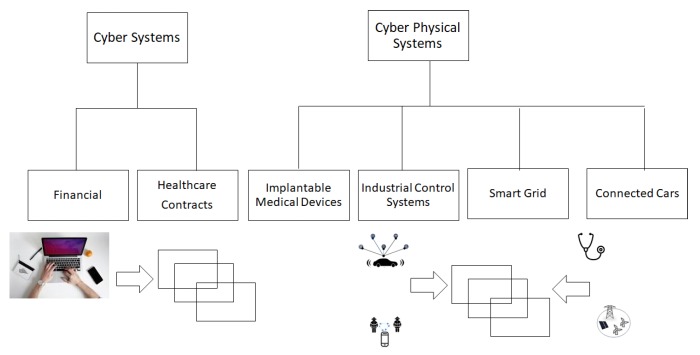
Four applications of CPS surveyed in this paper.

This is one of the many cyber applications, the ones enabled by computers. While such records are created and stored on computers, it still involves a human being entering the information. Financial transactions, health records, insurance records are some of the many examples in this category. So, one can say that humans were still the primary source of data collection in these applications. Over the past few years, fueled by the emergence of IoT and driven by the proliferation of sensing technology, sensors are now replacing humans as the primary source of data collection in many systems. Such systems, called CPS, combine physical processes, software, and communication to provide an integrated system with abstractions, design, and analysis capabilities. The technology spans research across multiple disciplines, having core components, such as embedded systems, real time communication, computer, networking, and physical systems dynamics. The use of blockchain for making a financial transaction has been well researched and documented. Advances in this technology have helped in sending money directly to the authorized people without including centralized authorities. Application of blockchain as smart contracts minimizes the possibility of delays, suppression, or any other outside influence. It applies comprehensive financial security, monitors the terms of the contract and is unbreakable. It also makes it easier to track and monitor digital identities using blockchain. The usage of blockchain as a cheap notary system has been described in [15], thereby avoiding different types of scams by creating unique certificates which would be easy to verify. In similar lines, a recent review of blockchain in education is given in [16]. This paper mainly focuses on an emerging application of blockchain for cyber-only systems, namely health records and four representative applications of CPS, namely implantable medical devices, industrial control systems, smart grid systems, and connected cars (Figure 6). Table 1 outlines the application domains of various systems discussed in the paper, along with the societal impact in each system.

### 3.1. Blockchain Applications in Healthcare

Blockchain is now being used for record management in applications, such as public health and medical research based on personal patient data. Evaluation metrics based on feasibility, intended capability, and compliance can be used to assess blockchain based decentralized applications in the are of health care [17]. The underlying benefit of blockchain, critical for health data, is that it is impossible to change or delete a record without leaving a digital trace of the attempt to do so. Many countries, such as Estonia, are using blockchain to secure the health and clinical trial records by linking access to data with permission settings. Blockchain also offers security through transparency, which enables the scanning of barcode-tagged drugs and helps them enter into secure digital blocks whenever they change hands, thereby reducing the chances of counterfeiting. This can be further secured by allowing only authorized parties at the far end of the supply chain to access the real time records. There are a diverse set of applications where blockchain can be utilized, namely data sharing, access control, health records, managing an audit trail, and supply chain [18]. Active scientific work being done in some of these areas is surveyed next.

#### 3.1.1. Healthcare Record Management

The management of the integrity of the healthcare records and clinical preliminaries is pivotal. From the time instance when a medical record is created and marked, administrators are required to maintain evidence that the same has not been modified illegally, thereby maintaining the sanctity of the record. The field of healthcare record management deals with interoperability, information exchange, and analytics. Healthcare interoperability, as defined in [19], can either be institution driven or patient driven. The shift towards the latter brings with it various difficulties related to patient consent governance, security, protection, and patient commitment. Many scientific papers have been published showing how blockchain can facilitate the administration of digital access rules related to information aggregation, information availability, and liquidity, as presented in [20]. Furthermore, it also helps with the understanding of patient attribute and it’s immutable nature. Scientific work on how a patient could safely collaborate with numerous stakeholders, recognize themselves over every entity and aggregate the health information using an abnormal structure in a persistent form is presented in [21,22]. A study of how interoperability is addressed among healthcare blockchain applications can be found in [23]. The MedRec [24] model gives a proof-of-framework, which enables standards of decentralization and blockchain designs to anchor and inter-operate across medical record systems. It uses the ethereum smart contracts to organize the framework and generates a log, which oversees medical records while giving patients the ability to survey complete records, audit care records, and share information. In this work, an inventive method for coordinating with suppliers’ current framework, organizing open APIs and system structure transparency, has also been presented. A method adapted to handle big personal health data using a tree-based approach is presented in [25]. To provide protection to cloud-hosted records, an initiative using blockchain technology is presented in [26]. BlockHIE [27] is another blockchain based platform for healthcare data which combines off-chain storage and on-chain verification to provide privacy and authentication to records. An architectural design, presented in [28], uses blockchain to facilitate healthcare data sharing in a private and audit-ready manner. It also handles permission-based healthcare data access using blockchain. Furthermore, a centralized source of trust in favor of network consensus and prediction of consensus of proof of structural and semantic interoperability is presented in [29,30]. Finally, in the area of healthcare analytics, a blockchain-based application for storing and managing the database of patients and doctors during surgery is stated in [31]. A framework, based on artificial systems, computational experiments, and parallel execution using blockchain technology, which brings in the benefits of a parallel healthcare system, is proposed in [32]. A proposal to manage health data at the individual and institution level using private blockchain solution has been made in [33].

#### 3.1.2. Implantable Medical Device Security

Smart systems allow persistent remote patient monitoring, thereby making it a prominent health-care technology [34,35,36], thereby making healthcare information as a valuable source of medical intelligence [37,38,39]. In the previous section, we looked at research centered around the management of medical information. In this section, we will study recent advances in the sharing of healthcare information which has the potential to significantly benefit the quality of healthcare data. According to statistics, the United States spends a bigger rate of the gross domestic products and more per capita on medical care (a record $2.5 trillion (17.3%) in 2009 spending around $8050 per individual) [40]. National well-being consumption is anticipated to grow at a normal rate of 6.3% every year through 2019, thereby achieving 19.6% of GDP by 2019 [40]. By the end of the year 2019, the market share for medical devices is expected to reach $186 billion, thereby making it one of the biggest markets in this space [40]. U.S. exports in medical devices, as recognized by the Department of Commerce (DOC), surpassed $44 billion in 2015 [41]; largely fueled by major innovation happening in more than 6500 medical device companies in the United States.

Table 2 presents the various blockchain use cases, design challenges, and future directions in healthcare.

A case study of blockchain and IoT powered healthcare is presented in [42]. An amalgamation of blockchain and IoT in healthcare can be used for collecting and processing data in real time and providing secure access and data exchange between care providers. Using a private blockchain based on the ethereum protocol, authors in [45] have used an ethereum-based private blockchain protocol, to create a smart contract, where the sensors communicate with smart devices. Such contracts manage records of all events on the blockchain to support real-time patient monitoring and medical interventions. MeDShare [43] is another trust-less system to share medical data using cloud service providers via blockchain. It has been shown to achieve data provenance and auditing while sharing medical data with diverse set of entities. Authors in [25] have designed a decentralized, permissioned blockchain for user-centric health data sharing. It has been designed to protect privacy using channel formation scheme and enhance identity management using the membership service in mobile healthcare applications. A system that ensures the security of patient’s data through self-management, thereby preventing privacy violation, is presented in [44].

### 3.2. Blockchain Applications in Industrial Control Systems

**Figure 7:**
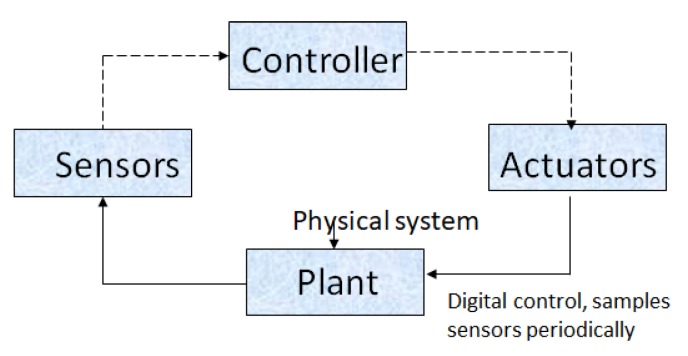
Industrial control systems.

Industrial control systems (ICS) refers to such systems that control and monitor the physical entities that can be used in a diverse set of industries, ranging from mission-critical nuclear plants and commonplace irrigation systems. ICS senses and collects data through sensors and passes the information to the controller, which in turns sends the feedback through the actuator, as shown in Figure 7.

The key components in the ICS environment are:

- A plant capable of data acquisition, communication, and local processing, using operational technology. This is called as sensors. It is a device which measures physical quantity. Examples of sensors are cameras, accelerators, gyroscope, Lidar, Radar, etc.
- A computing device, typically referred to as Programming Logic Control (PLC), that can be programmed to perform operations based on programmable logic. It has been traditionally used both in Distributed Control System and Supervisory Control and Data Acquisition systems to control the overall system. A data historian is maintained on the controller to log information related to all the process. The same can be used for algorithms, parameter configuration, monitoring, and set-point configuration.
- A control loop that enables the controller to execute on different tasks by interpreting sensor signals. Actuators are a part of this system, which modifies the physical quantity observed by the plant. Typical examples of actuators are motor controllers, LEDs, lasers, loudspeakers, switches, valves, etc.

The Industrial Internet of Things (IIoT) is a significant component of the future transformation of industrial systems. Similar to ICS, the interconnection and intelligence is provided through sensing devices and actuators with ubiquitous networking and computing capabilities. It is speculated that by the year 2020, billions of devices capable of generating data will be connected to the internet. This will benefit various applications, such as infrastructure, transportation, and agriculture. In such systems, transactions include readings of data acquired from various sensors with a spatio-temporal stamp indicating where and when the reading was taken. Such data is then shared among various players in the network. Similar to the concept of financial transactions, it is critical to maintaining a historical record of these transactions as they are used to impact mission critical decisions. This mandates that the records are not tampered with illegally and a trace is maintained in case such attempts are made.

Blockchain introduces a robust and efficient next generation of techniques for the transactions generated by the physical resources. The mix of blockchain and IoT gives us a versatile, truly distributed, peer-to-peer system and the capacity to interact with distributed sensors in a trust-less, auditable way. A framework using ethereum to communicate electricity usage, including air conditioners and bulbs, was proposed in [46]. The ethereum notifies the network to update devices from normal to energy saving mode to make efficient usage of the electricity. Another such effort for making smart homes more efficient by significantly lowering overhead due to traffic, processing time and energy consumption was presented in [47]. Furthermore, to support fast and secure energy trading in IoT applications, a consortium of blockchain providers is proposed in [48]. The authors propose using available information and energy interaction perspective to create a network that can make decisions that are context-aware. A technique based on distributed consensus to obtain proof of work, using the frequency of data and the amount of energy contribution, is presented in [49]. The work in [50] presents the usage of blockchain technology as a platform for hierarchical and distributed control systems based on the IEC-61499 standard. In this work, Hyperledger Fabric, where functional blocks are implemented as smart contracts on a supervisor level, was selected as the blockchain solution.

A blockchain-based system for secure mutual authentication, to enforce access control policies is discussed in [51]. This system uses a triangulation with integrated attribute signature, multi-receivers encryption, and message authentication code and is designed to provide privacy and security guarantees. Another work along similar lines is presented in [52], wherein the authors use blockchain to create virtual zones to present a robust method for identification and authentication of devices. These virtual zones form a distributed system called Bubble of Trust, which ensures a robust identification and authentication of devices and protects the integrity and availability of the data. Authors in [53] analyzed unique functions and open challenges of blockchain, as well as discuss a potential application that stands at the intersection of blockchain and IoT. A fully distributed access control system for IoT based on blockchain technology, used for arbitrating roles and permissions in IoT, is discussed in [54]. A blockchain-based privacy protection management scheme for IoT devices, which combines attribute-based encryption with time-limited key management technology to achieve privacy protection and device management, is described in [55]. Authors in [56] propose a distributed fair access control framework based on cryptocurrency. It provides granular level access to data through smart contracts by using the consistency of blockchain technology to manage access control on behalf of constrained devices. A theoretical lightweight architecture based on private blockchain in the context of smart home, which reduces the communication overhead of workload proof mechanism by introducing the central miners, is discussed in [57]. Table 3 presents the use cases, design challenges, and future directions in ICS.

When all the devices in an IoT network are connected to each other and have decision-making capabilities, one can automate time- and human resource-consuming work processes. However, this mandates the need to maintain a historical ledger of these actions and the data that led to the actions. Active scientific research, such as the one cataloged above, indicates that the incorporation of blockchains in the IoT area will fulfill the need for cryptographic verifiability, thereby affecting critical changes over several industries.

### 3.3. Applications in Transportation

**Figure 8:**
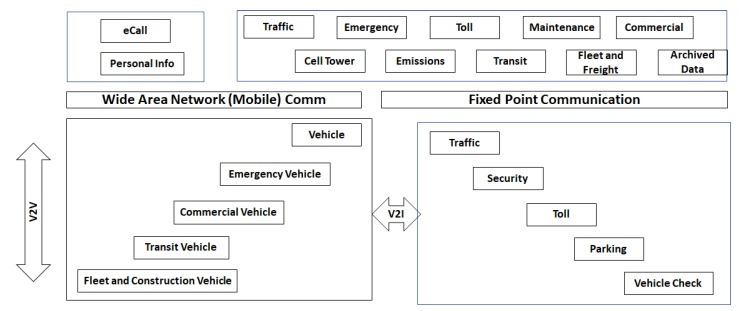
United States Department of Transportation (US DOT) Intelligent Transportation Systems (ITS) National Architecture.

Autonomous vehicles are the future of transportation and will play a crucial part in how society evolves. These vehicles play an important role in improving the connectivity and providing road safety, better traffic management and driver comfort. Blockchain can be used to build up a verified, trusted and decentralized self-governing intelligent transport system, making better use of the heritage intelligent transport systems (ITS) framework and assets, particularly successful for crowd-sourcing of innovation. Figure 8 presents the ITS national architecture proposed by department of transportation. ITS-oriented, a seven-layer conceptual model for blockchain is proposed in [58]. The seven layers are physical, data, network, consensus, incentive, and application layer, respectively. Additionally, a distributed key management in heterogeneous intelligent transport systems is proposed in [59]. It includes the key transfer between heterogeneous networks and the dynamic key management scheme to decrease the key transfer time.

Refueling scenario for autonomous electric vehicles using blockchain to guarantee the execution of energy recharges is discussed in [60]. A reward-based intelligent vehicle communication based on blockchain technology is presented in [61]. It improves the privacy and provides fast, secure communication between vehicles.

Connected cars have built-in sensing features, through which they monitor their surroundings and build a comprehensive 360-degree view of what is around them. For instance, they have built-in navigation systems, cameras, proximity detection sensors, light and radio-frequency detection sensors, to name a few. They have the ability to synchronize information from multiple sensors, a technique known as sensor fusion, with real-time data to keep the vehicles and infrastructure elements informed, in case of accidents. Such features have led to an increase in the number of advanced driver assistance functions, such as adaptive cruise control, lane change warnings, and collision avoidance mechanisms. To enable all of these functions, such cars are also equipped with communication devices and protocols that are used to share information between all the entities in the vehicular network. Dedicated Short Range Communications (DSRC) is the currently approved protocol for 5.9 GHz Intelligent Transportation Systems (ITS) band to handle Vehicle to Vehicle (V2V) safety applications [62]. It uses IEEE 1609.2-4 message protocol and security services to enable use-cases for V2V Communication, which includes emergency electronic brake lights, forward collision warning, blind-spot detection. Information is shared between vehicles using SAE J2735 Basic Safety Messages (BSMs). BSMs provide position, size, velocity information to other vehicles, thereby creating awareness about the environment. Such information is encrypted using PKI-based certificates, thereby ensuring that the safety message is from a trusted source. For V2V communications, basic safety messages are trusted but not encrypted because they are broadcast to all the neighboring vehicles, while certificate messages are both trusted and encrypted. While BSMs have built-in encryption, it has been demonstrated that they can be tampered with, thereby leading to serious safety and security concerns. As an example, Charlie Miller and Chris Valasek have demonstrated attacks on a Toyota Prius and a Ford Escape using simple off-the-shelf components, such as Uconnect head unit, which is used for remote access [63]. Likewise, researchers at the University of South Carolina, Chinas Zhejiang University, and the Chinese security firm Qihoo 360 demonstrated that they could jam such sensors from a popular electric vehicle, thereby making objects invisible to its navigation system. They were able to precisely jam the radio signals by simply by using two commercially available off-the-shelf radio equipment and basic signal generation and frequency control instruments [64]. It is conceivable that the units inside the vehicle, such as engine control unit, brake control unit, and wheel control unit, are also vulnerable to such malicious attacks since they are also heavily dependent on communication. While the occurrence of such attacks has been largely limited to date, with the drastic increase in the number of connected vehicles, the surface area of such attacks is bound to increase much. It is indisputable that security in such types of vehicles is imperative and critical.

While Public Key Infrastructure (PKI) today handles the security aspects of these messages, it suffers from the same limitations as any centralized authentication system. Additionally, a centralized PKI lacks true information about ground reality as it does not have the sensing capabilities available on vehicles. Blockchain holds a cutting edge potential for these cars. In the case of connected cars, the transactions shared between vehicles are the basic safety messages, which contain information about the size, position, velocity and heading of the car. These messages are digitally signed and the signature is validated by the PKI. They have to be maintained in a time sequenced historical manner in the folders for use-cases related to law enforcement and insurance claims. It is critical that these transactions are validated in real-time for immediate use-cases related to higher levels of automated driving. Additionally, these transactions should not be tampered with any time in the future, as they may be needed for judicial and insurance claim reasons. All of these requirements, make blockchains a viable option to consider for transaction management in connected vehicles.

Authors in [65] proposed a decentralized technique to protect against attacks on sensors and communication channels. They do so for securely sharing messages between connected vehicles based on a blockchain architecture. Trust bit [66] uses the blockchain approach to implement intelligent vehicle communication using a reward based scheme. It exchanges trust bits as rewards during successful communication. For recording and maintaining historical evidence of transactions involving such trust bits, they used blockchain technology in the vehicular cloud. This allows all the trust bit details to be securely accessed by vehicles independent of space and time constraints. The concept of a local dynamic blockchain and main blockchain has been explored in a branch based blockchain technology presented in [67]. The underlying new idea is the definition of a secure and unique crypto ID, called an intelligent vehicle trust point, to ensure trustworthiness among vehicles. Vehicles use the local dynamic blockchain to verify the IDs while they are communicating with other vehicles. A new secure blockchain-based communication scheme for connected vehicles is presented in [68]. In this scheme, the identity of the vehicles joining the network is first verified by a ring-signature based scheme. Next, the consensus among the vehicles is achieved using a blockchain-based mechanism prior to sharing the information, created by multi-party smart contracts, using secure communication channels. A blockchain technology which uses multi-signature mechanism proposed in [69]. It provides emerging vehicular services, such as remote software updates, without revealing any of the vehicles’ private information. Yuan et al. [58] proposed a seven-layer conceptual model for intelligent transportation using blockchain technology, thereby creating a secure and trust-worthy decentralized ecosystem. Leiding et al. [70] have combined ethereum-based smart contracts with vehicular network technology. As vehicles are increasingly becoming software-dependent, a key question which must be answered is about updates to the software as new features are added. A design of how blockchain technology can be used to do this has been shown in [71]. In this approach, an overlay network is used to transfer messages between software providers, cloud storage mechanisms, and vehicular interfaces. Such messages are used to initialize the blockchain system and handle the software distribution processes. Vehicle system with vehicle report generation and methods for use are presented in [72] a processor is configured to perform data-driven operations, such as report generation. Such operations are generated using a vehicle-specific digital currency record using cryptocurrency protocol. The value of this digital currency is adjusted based on the price of goods or services purchased. It is stored in memory and communicated while the purchase of goods or services is in action. Using visible light and acoustic side-channels, Rowan et al. [73] proposed a new blockchain technology for securing communication in cars. Table 4 presents the use cases, design challenges, and future directions in transportation sector.

### 3.4. Applications in Smart Grid Systems

**Figure 9:**
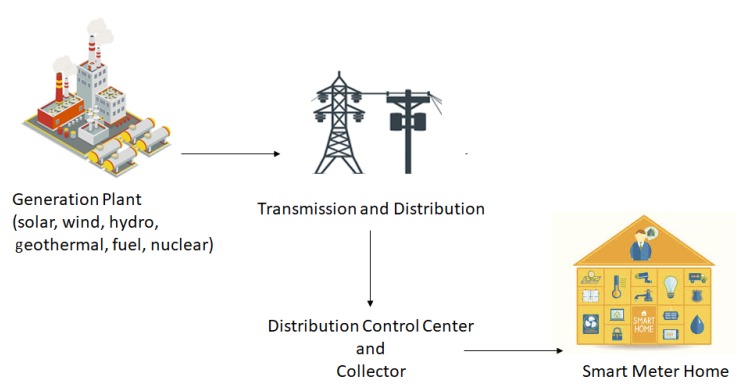
Smart grid architecture.

Access to electricity is a fundamental need for modern society and the economy. An estimated $48 trillion investment is required in the energy infrastructure over the period of the next fifteen years [74]. This poses an imminent need and opportunity to shift towards an efficient and clean energy system with a low carbon footprint. Smart grid systems play the role of a necessary enabler for this transition. A smart grid is an intelligent, digitized energy network delivering electricity in an optimized way between source and consumption, as shown in Figure 9. This is accomplished by integrating information, telecommunication, and power technologies with the existing electricity system. Smart grid systems incorporate sensors and software on the existing grids, thereby equipping utilities and personal users with information that enables them to react to changes quickly. In addition to improvements in the efficiency and reliability of electricity supply, smart grids play a catalyst role in the integration of renewable energy into existing networks, thereby reducing carbon emissions.

Different ways in which blockchain can be used to modernize the grid have been discussed in [75]. A framework for information exchange and buy-sell transaction mechanisms between energy providers and citizens using blockchains is proposed in [76]. Existing power grids do not provide resilience against cyberattacks on distributed energy resources and grid edge devices. Authors in [77] have discussed a business model to reduce costs by cutting out third parties. They also discuss techniques to increase arbitrage opportunities to produce and sell energy at an individual level. In [78], energy usage information is collected in a distributed fashion from smart metering devices. The expected energy flexibility at the consumer level can be controlled in a programmatic manner using self-enforcing smart contracts. By tracking the flexibility between energy consumption and demand response signal, authors have shown how energy demand and production can be matched at a smart grid level. They have also combined the reward and penalty mechanism to balance energy demand. Blockchain based smart contracts presented in [79], provide security and resilience, an immutable transaction history, and the ability to enable transactions, and automation at a micro-level in an effective and profitable manner. Authors in [80] present a local energy market design and simulation, implemented on a private blockchain with artificial agents, to offer real time pricing information. It simulates optimum decisions based on production capacity prediction, thereby automating informed tariff decisions. Similar work, wherein production and consumption load profiles are transformed in a distance-preserving embedding in order to find a matching tariff, is presented in [81]. It uses blockchain to make the calculations for tariff matching publicly available, while still maintaining the privacy through embedding. This work has been further extended and validated on electric vehicles [82]. A similar work, where a blockchain-based privacy preserving payment mechanism is proposed for a vehicle to grid networks, enables data sharing while securing sensitive user information [83].

A modular platform-based approach for applying cryptocurrency features to the renewable market has been shown in [84]. This work also includes a robot which advises users on the best selling strategy. A smart replicable district model which uses new technology to build an efficient energy management system integrated into a platform based on an IoT and blockchain approach is presented in [85]. A blockchain-based method for power grid communications in smart communities which preserves privacy and manages efficient aggregation is proposed in [86]. Here, a blockchain based solution is proposed to avoid application usage patterns by analyzing the user’s electricity consumption profile. Along similar lines, a sovereign blockchain which provides transparency and provenance is utilized to mitigate the security and privacy concerns on smart grid [87]. A proof-of-concept for decentralized energy trading system using blockchain technology, multi-signatures, and anonymous encrypted messaging streams, enabling peers to anonymously negotiate energy prices and securely perform trading transactions is presented in [88]. A control strategy using proportional fairness to create incentives for distributed energy resources to operate at sub-optimal capacity has been described in [89]. In this scheme, a subset of the network actors decreases their power output and revenue to aid the overall system performance. A historical track record of these transactions is managed using blockchains. Table 5 presents the use cases, design challenges, and future directions in smart grid domain.

## 4. Blockchain Limitations and Future Directions

As demonstrated by the expanse of the scientific research surveyed in this paper, blockchain technology has been gaining rapid popularity in recent years. It has the potential for changing the way in which people work and communicate by laying the foundation for emerging applications using connected devices. However, it has certain limitations, such as:

- It does not scale with the number of connected devices as it is limited by its usage of block size and time needed for hash calculations.
- In some cases, it mandates the need for transaction fees or some other reward mechanism for miners.
- While it is not as centralized as the concept of a single bank, it is still dependent on a handful of big entities, such as miners.
- The computational and storage requirements of the blockchain participants are extensive since they have to store the entire ledger, and they participate in the transaction verification process as endorsers, or miners.

**Figure 10:**
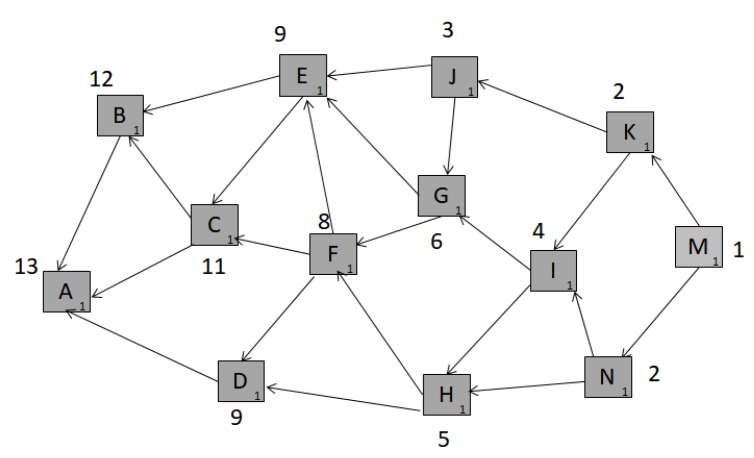
A snapshot of tangle with direct and cumulative weight of the sites. The boxes represent transactions; the small number in the corners of each box denotes own weight, and the bold number on top denotes the cumulative weight.

These limitations make blockchain not ideal technology for very a large IoT system of connected devices. Due to this, in 2017, tangle was presented as a technology for transaction validation and security for the Internet of Things (IoT)-related applications [90]. It is better suited to meet IoT requirements, such as low resource consumption, widespread interoperability, billions of nano-transactions, and data integrity, as it is faster, energy and resource-efficient, and quantum-proof. Tangle is a progressive transactional system and information exchange layer, designed for securing applications around the IoT. It is based on a directed acyclic graph called tangle, which is a typical data structure technique. It is designed to overcome some of the inefficiencies of blockchain. In the tangle network, each transaction needs to validate two previous transactions by conducting a PoW. The underlying theory is that the network will scale faster as more transactions are being validated in parallel. Tangle has properties of scalability, resource optimization, data transfer security, and quantum readiness. As shown in Figure 10, in a directed acyclic graph box are the sites (or transactions). The edges represent the link connecting the transactions which validate prior transactions. In order for a new transaction to be considered as a part of the network, the issuer has to verify two unconfirmed transactions, referred to as tips for the tangle. In addition, the new transactions have to perform three basic functions in order to be considered in the network, namely:

- It has to sign its transaction to authenticate itself in the network.
- It has to randomly select two, non-conflicting, transactions to validate.
- It has to do work to validate these selected transactions, which is similar to PoW in blockchain.

There are typically two kinds of weights in tangle: direct weight and cumulative weight.

**Figure 11:**
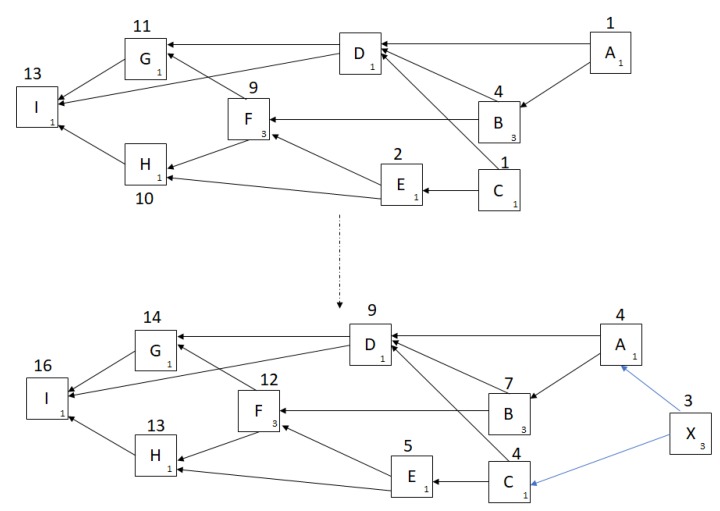
Weight assignments before and after a newly-issued transaction, X.

The direct weight of a node is generally expressed as 3 raised to a power of any real number. On the other hand, cumulative weight is obtained by adding the direct weight of all the sites which has directly or indirectly verified the previous site. Figure 11 shows weight assignments before and after a newly issued transaction, *X*. The first part is the snapshot of tangle at a particular time instant. *X* when issues a transaction, selects two non-conflicting transactions using Markov Chain Monte Carlo technique [91,92]. For instance, if it selects transaction *A* and *C*, the cumulative weight of all the nodes is updated by 3. This is because it directly or indirectly affects the overall network structure.

Tangle is being presented as a third generation cryptocurrency that does not have the overhead of transaction validation but is nevertheless secure. Table 6 compares blockchain with tangle.

Tangle is more decentralized than blockchain. Blockchain will probably connect multiple IoT devices to one gateway and then the gateway would be a participant in the blockchain network; we can call this a clustered or a semi-decentralized approach. It promotes the concept that the lightweight IoT device can be a participant in the tangle network directly.

## 5. Does Your Application Benefit from Blockchain?

The requirements of many applications are adequately met by traditional relational databases; and as such these applications do not benefit by using a decentralized database, such as blockchain or IOTA. Since there are significant cost and performance trade-offs while using a decentralized database, it is critical that a decision support system (DSS) is used to make this decision. In this section, we present a mathematical model for such a DSS. While it is generally considered that all models are wrong but some are useful, as presented by G.E. Pelham Box in [93], we hope that the model presented in this paper serves as an useful tool to aid in decision making. The mathematical model is defined using the following equation:

(1) $$s=\frac{\sum_{i=1}^{N}a_{i}w_{i}}{N},$$

where

- *s* is the overall score,
- $w_{i}$ is the weight for the metric under consideration, 0.0≤w≤1.0,
- *N* is the number of metrics under consideration, N>1, and
- $a_{i}$ is the scaling factor for the metric under consideration, 0.0≤a≤1.0.

Users can analyze the output of this model, the overall score *s*, using the cost-benefit analysis principles [94] and determine if a particular application can benefit from a decentralized database. The value of the scaling factor *a* is generally a complex function of the specific metric *m* being considered. There are two generally-established techniques in which the value of *a* may be determined. The first approach is to perform a complex mathematical analysis of the benefits associated with using the decentralized database for a particular application. A second approach is experiential in nature and uses a semi-structured survey-based approach to gather participant inputs on the topic. The objective of such a survey is to collect the respondent’s views on the importance of a metric for their application. A semi-structured approach to gather participant’s feedback on a particular topic has been described in [95]. Such an approach has been used to gather participant feedback in the evaluation of novel stroke rehabilitation technologies in [95]. Likewise, this approach has been used in a European Union (EU) report on coordinated risk assessment of cybersecurity in 5G networks, published by the NIS Cooperation Group [96]. In this report, participants from member states submitted their input on national risk assessments based on a questionnaire, based on the results of their national 5G cybersecurity risk assessments. The participant list included cybersecurity and telecommunication authorities, security, and intelligence services. The information provided by the member states allowed the collection of information on main assets, threats and vulnerabilities related to 5G infrastructure and main risk scenarios, describing potential ways in which threat actors could exploit a certain vulnerability of an asset in order to impact government objectives.

In this paper, using a similar approach, we created a questionnaire which assesses the applicability of a decentralized database for a particular application, based on a set of five metrics as described below:

- *Multiple Writers*: This metric considers the probability whether there will be multiple writers to the database. The participants were asked “What is the likelihood (probability) that the records in the database be entered by multiple writers? 0 implies it is highly unlikely, 1 implies it is highly likely?”.
- *Rogue Untrustworthy Actors*: This metric considers the probability whether there will be rogue actors in the database who can disrupt trust in the system. The participants were asked “What is the likelihood that there can be rogue/untrustworthy actors in the system? 0 implies highly unlikely, 1 implies highly likely?”.
- *Scalability*: This metric considers the need for scalability in the system and how the database architecture can adapt to an increased number of nodes in the system, without incurring additional overhead, cost, or bandwidth constraints. The participants were asked “What is the likelihood that the system needs to be scalable? 0 implies highly unlikely, 1 implies highly likely?”.
- *Historical Transaction Ledger*: This metric considers the need for maintaining a historical ledger of transactions. Such a historical ledger could be required for governance and policy requirements and is typically found in banks, insurance companies and other highly regulated industries. The participants were asked “What is the likelihood that the database needs to keep a historical ledger of all transactions? 0 implies highly unlikely, 1 implies highly likely?”.
- *Security*: This metric considers the level of security needed in the database. While security is paramount for all application, this metric considers the relative importance of security for one application over the other. The participants were asked "How critical is it to maintain security in the system? Enter a number between 0 to 1, 0 indicates not critical, 1 indicates most critical" The format of this question is different from the first four, but we have kept the score range consistent to fit the model.

We conducted a survey where participants were asked to evaluate the applicability of distributed database for two applications, namely a university database that maintains students grades, henceforth referred to as A1, and a database that maintains basic safety messages between connected vehicles, henceforth referred to as A2. We chose these two applications for the following reasons:

- They represent applications that are practical and well-understood for the survey participants.
- They represent applications where security is a strong requirement.
- While application A1 represents lower end of the complexity spectrum, application A2 represents upper end of the complexity spectrum.

Students answered the above questions by giving a score between 0 and 1, as described above. It is important to note that the questions were presented such that the participants understood that they are comparing applications A1 and A2 for the decentralized database only. The survey was undertaken by 105 participants, who were database science practitioners enrolled in the Master of Science in Business Analytics (MSBA) course at the University of Texas at Austin [97]. Participants had a working knowledge of relational databases (such as SQL), big data processing using Hadoop and Spark, and decentralized techniques, such as blockchain and tangle.

**Figure 12:**
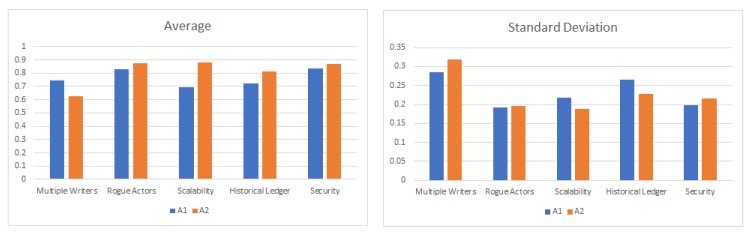
Average and standard deviation plot. Participant’s score for the five questions for applications A1 and A2.

Figure 12 shows the average and standard deviation of the participant scores for each of the five questions for both the applications A1 and A2. Assuming that the weight for all the metrics is 1.0, if we plug the above values in Equation (1), we get the overall score of 0.80 for the university database application, versus an overall score of 0.81 for the connected vehicle database. Using this framework, we can determine that a distributed ledger based decentralized database technique is equally well-suited for both the databases that maintain basic safety messages in a connected vehicle versus a university database of student scores. Table 7 compares A1 with A2 on various metrics.

**Figure 13:**
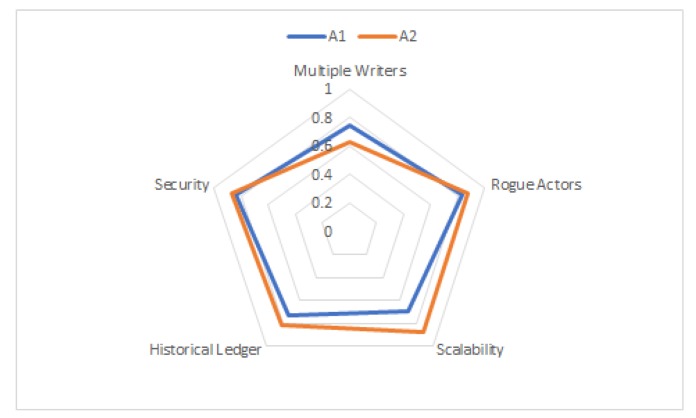
Radar plot: A visual tool for comparing the effectiveness of using a decentralized database for two different applications A1 and A2. A1 is the University Database, and A2 is the Connected Vehicle database.

While the mathematical model described above provides a simple numerical output for decision making, in some situations, a radar plot is a powerful visual tool that can be used to compare the applicability of decentralized database for different applications, such as the university database (A1) and connected vehicle database (A2), in this case. A radar plot is particularly useful in situations where there are large numbers of independent variables, possibly with different measurement scales. This approach is particularly used in areas where researchers wish to illustrate the degree of multiple-group consensus or group differences on multiple variables in a single graphical display. Radar plots have been used in number of applications, such as multivariate healthcare data [98], multivariate data analysis [99], and nondestructive quality evaluation of manufacturing processes [100]. Using wheel taxonomy, a spoke is one of the many rods radiating from the center of a wheel, connecting the hub with the round traction surface. A radar plot consists of a sequence of such equal angle spokes, with each spoke representing one of the variables. The data length of a spoke is proportional to the magnitude of the variable for the data point relative to the maximum magnitude of the variable across all data points. A line is drawn connecting the data values for each spoke. This gives the plot a star-like appearance. A radar chart is a useful visual tool to determine which observations are most similar, i.e., identify if there are clusters of observations and identify if there are outliers. It also allows us to explore hybrid mechanisms for database design, which combines the strengths of relational and distributed databases. Figure 13 shows a radar plot of the average value of the survey score for the two applications we considered above. The spokes in this radar plot represent the five metrics chosen as part of the survey questionnaire. While the area under the curve is almost equal for both the applications, the radar plot clearly shows that the decentralized database approach has a higher score (hence, better applicability) for the connected vehicle database for the metrics of scalability and maintaining historical transactions.

## 6. Conclusions

This paper provides a holistic survey of various CPS where decentralized database techniques, such as blockchain or tangle, are used. CPS are systems that control and monitor the physical world around us. Blockchain and their inherent combination of consensus algorithms, distributed data storage, and secure protocols can be utilized to build robustness and reliability in these systems. This paper describes how applications, such as a smart grid, autonomous vehicles, and IoT devices, have benefited by distributing the role of information validation across the network peers, thereby eliminating the risks associated with a centralized architecture. This paper surveys advancements, use cases, design challenges, and future directions in blockchain research across the healthcare, smart grid, autonomous vehicle, and industrial production process applications and demonstrates how these applications have benefited from this technology. This paper describes blockchain technology, which is a shared database that grows only by appending new data, authenticates users with strong cryptography, and leverages economic incentives to encourage mistrustful strangers to manage and secure updates. This survey paper presents the advantages and disadvantages of this revolutionary technology. This paper also describes a mathematical model that can be used as an aid to determine if a particular application can benefit from this technology. The model was tested on two applications, namely the connected vehicles database and university database. The study concludes that the decentralized database is valuable for both of the applications. For future work, we plan to conduct similar study on other applications, such as smart grids, healthcare, and IoT devices.

## Figures and Tables

**Table 1:** Application domains of CPS.

Systems	Applications	Societal Impact
Healthcare	Medical devices, health management networks	World class medicine and health care systems
Transportation	Automotive electronics, railroad systems, vehicular networks, aviation and airspace management	Zero automative traffic fatalities, reduced traffic congestion and delays
Industrial Control Systems	Physical infrastructure monitoring and control	Maximum yield and performance
Smart Grids	Electricity generation and distribution, building and environmental control	Blackout free electricity and distribution, environmental benefits

**Table 2:** Blockchain use cases, design challenges, and future directions in healthcare.

Application Domain	Objectives/Use Cases	Future Directions
Healthcare interoperability [19,21]	Data exchange, interpretation, and usage	Advanced data analytics, and supported by robust care coordination
MedRec record management [24]	Governs medical record access while providing patients with comprehensive record review, care auditability, and data sharing	Gather custom integration requirement to build open standard
BlockHie [27]	Healthcare information exchange for electronic medical records and personal healthcare data	Off-chain storage and on-chain verification for privacy and authentication
Healthcare analytics [31]	Acquisition, storage, and sharing of health data	Blockchain with artificial intelligence for healthcare analytics
Blockchain and Internet of Things (IoT) powered [42]	Big data incorporation for data mining	Require consensus model, less computational costs for mining blocks, and validating transactions
MedShare [43]	Data sharing model between cloud service providers	Decrease latency contributing to the processing and anonymization of data
Data sharing and Privacy [25]	A tree-based data processing based on Hyperledger fabric and batching method to personal health data	Combine both personal health data and medical data together
Privacy Violation [44]	Anonymization, communication, and data backup and recovery	Secure raw data rather than anonymizing

**Table 3:** Use cases, design challenges, and future directions in industrial control systems.

Application Domain	Objectives/Use Cases	Future Directions
Managing IoT Devices [46]	Save data coming from meter and smart phone	Requires large storage, not time efficient
Smart Home [47]	Lightweight security, symmetric encryption employed for smart home	Explore applications in other IoT Domain
Secure Energy Trading in Industrial Internet of Things (IIoT) [48]	Maximize economic benefits of credit banks	Schemes designed for extreme scenarios with excellent or poor credit values
Electric vehicles cloud and edge [49]	Data contribution frequency and energy contribution are applied to achieve the proof of work	Hybrid cloud computing and edge computing for center-less trust, collaborative intelligence, and spatio-temporal sensitivity
Distributed control system for edge computing [50]	Higher level performing supervision and strategic decisions and lower level having direct control of devices and processes	Executive level responsible for process control
BSeIn [51]	Secure mutual authentication with access control for industry 4.0	Integrating intra-organizational value networks
Bubbles of trust [52]	Secure virtual zones where things can identify and trust each other	Cooperation between virtual zones
Blockchain meets IoT [54]	scalable access management in IoT	Requires adaptable technology for IoT scenarios
Device management scheme on blockchain [55], privacy preserving [56]	Sharing of device information without breaching confidentiality	Possibility of anonymizing the data, other challenges and solutions include fault tolerance, policy enforcement, non-reputation, trust [57]

**Table 4:** Use cases, design challenges, and future directions in transportation.

Application Domain	Objectives/Use Cases	Future Directions
Intelligent transport systems [58]	Seven layer conceptual model for intelligent transport systems	Explore the rationale, novel business models, as well as practical application scenarios
Distributed key management [59]	Uses the dynamic transaction collection period to further reduce the key transfer time during vehicles handover	Pseudonym management using blockchain
Charge it up [60]	State Channel for smart mobility systems for delay, latency, security, and cost	Smart mobility systems can use state channels for control logs and connectivity
Reward based systems [61]	Trustworthiness for vehicles behavior, and vehicles legal and illegal action	Multiple vehicle action for suspicious scenario.
TangleCV [65]	Distributed trust system for security	Vehicles moving in and out of network
Trustbit [66]	Intelligent vehicle communication using a reward based scheme	More use cases on communication level
Intelligent vehicle trust point [67]	Crypto ID to ensure trustworthiness in vehicles	Usage of bitcoin for paying on the gas stations
Identification of vehicles [68]	Secure blockchain-based communication	Perform moderate costly hash operations for the blockchain verifications.
Software update system [71]	Secure wireless (SW) update system	Validate the results on larger dataset
CUBE [69]	Network security platform	Use artificial intelligence (AI) to protect against malicious attacks

**Table 5:** Use cases, design challenges, and future directions in smart grid systems.

Application Domain	Objectives/Use Cases	Future Directions
Modernize Grid [75]	Industry flow, asset management, identity management, and smart contracts	The system should be less centralized
Smart energy grid [76]	Buy/sell energy between energy providers and private citizens	Citizens in the rural areas should be taken into account
Smart grid resilience [77]	Record real time loads and smart contracts execute customers distributed generated sales and purchases	Simulate applications in a realistic environment
Decentralized management of demand response [78]	Consensus based validation for matching energy demand and production	Implementation of multi-stakeholder markets
Blockchain based smart contracts [79]	Decreased payout times, reduced need for intermediaries	Microgrids will increase the resilience of the energy systems
Privacy Preserving smart grid tariff decisions [81]	Ensures transparency, verifiability, and reliability	Implementation in solidity
Electric vehicle charging [82]	Determine the cheapest charging station within a region	Scalability issue on large number of electric vehicles and handling the payment phase
Payment mechanism for vehicle to grid networks [83]	Data sharing and privacy protection in vehicle to grid networks	Diverse privacy demands, pricing policy
Crypto-trading energy market [84]	Robo-advisor to optimize the energy trading	Energy consumers to digitally connect to smart grid systems
Smart city through IoT [85]	Decentralized storage to record all transaction data	Replication in multiple cities
Efficient Aggregation for power grid communications [86]	Increased computational efficiency to preserve users privacy	Reduce the computational overhead caused by authentication, especially during system initialization
Grid-monitoring [87]	Prototype that allows user to monitor the electricity and no manipulation from the third party	Implementation of proposed model

**Table 6:** Blockchain and Tangle comparison.

Blockchain	Tangle
Blockchain is comprised of a series of nodes, or blocks of transactions, each one appended to the previous one in a long regularly-developing chain. It can loop back in circular fashion.	Tangle is comprised of a group of data nodes that only flow in a single direction. It can never loop back.
Decentralized with semi-distributed ownership.	Decentralized with truly distributed ownership.
Blockchain boasts a significant level of security, due to its block-formation process, which includes the solution of a mathematical problem and verification through group consensus.	Tangle only requires that a device validate and approve two previous transactions before it can finish one of its own and accordingly create a data node. This less-robust procedure renders the tangle less secure than blockchain.
Transaction speed declines as the network increases in size as more transactions compete for limited block spaces. This makes blockchain consume high computational power.	Tangle scalability increases as the number of users increases, which makes it lightweight; in turn, it requires low computation power.
High power leads to high energy requirements.	Low power consumption leads to less energy requirements.
Blockchain takes approximately 10 minutes to confirm a transaction, which makes it not scalable.	Since it has low overhead PoW, it is faster, which makes it more scalable in comparison to blockchain.
Miners take transaction fees.	Since there is no concept of miners, there are no transaction fees associated.
Not quantum resistant because it uses elliptic curve signature scheme.	Quantum computing protection because it uses hash based signatures.

**Table 7:** Metric comparison for A1 and A2.

Metric	A1	A2
Multiple Writers	0.75	0.63
Rogue Untrustworthy Actors	0.83	0.87
Scalability	0.69	0.88
Historical Transaction Ledger	0.73	0.81
Security	0.84	0.87
